# Molecular Characterization of Cocarriage Carbapenemase Genes in Clinical *Pseudomonas aeruginosa*

**DOI:** 10.1155/ijm/5679086

**Published:** 2026-10-02

**Authors:** Pompy Dey, Sudip Kumar Chowdhury, Ayesha Ahmed Khan, Md. Fourkan Uddin, Swagata Nandy, Shawon Banerjee, Puja Mitra

**Affiliations:** ^1^ Department of Microbiology Rangamati Medical College Rangamati Bangladesh; ^2^ Department of Community Medicine and Public Health Rangamati Medical College Rangamati Bangladesh; ^3^ Department of Microbiology Institute of Applied Health Sciences (IAHS) Chattogram Bangladesh; ^4^ Department of Microbiology Gopalganj Medical College Gopalganj Bangladesh; ^5^ Department of Microbiology Chittagong Medical College Chattogram Bangladesh cmc.edu.bd; ^6^ Anowara Upazila Health Complex Chattogram Bangladesh

**Keywords:** Bangladesh, carbapenemase, CAT, cocarrying carbapenemase genes, phenotypic vs. molecular

## Abstract

**Background:**

*Pseudomonas aeruginosa* is a major nosocomial pathogen associated with multidrug resistance and limited treatment options. Carbapenem resistance mediated by carbapenemase genes is an increasing threat, yet molecular epidemiological data from Bangladesh remain scarce.

**Objectives:**

To determine the prevalence of carbapenem resistance and carbapenemase genes, characterize cocarriage of carbapenemase determinants, evaluate phenotypic and molecular detection methods, and identify factors associated with carbapenem‐resistant *P. aeruginosa*.

**Methods:**

A cross‐sectional study was conducted from January to December 2023 at a tertiary‐care teaching hospital in Chattogram, Bangladesh. Clinical specimens from 335 hospitalized patients were cultured, yielding 48 PCR‐confirmed *P. aeruginosa* isolates. Antimicrobial susceptibility testing was performed using CLSI guidelines. Carbapenemase production was assessed by modified carbapenem inactivation method (mCIM). Multiplex PCR was used to detect blaNDM, blaVIM, blaKPC, blaOXA‐48, and blaIMP genes. Selected amplicons underwent Sanger sequencing and phylogenetic analysis.

**Results:**

Among 48 isolates, 27.1% were resistant to meropenem and 14.6% to colistin. Carbapenemase genes were detected in 20 (41.7%) isolates, with blaNDM being the predominant determinant (22.9%). Three isolates exhibited cocarriage of carbapenemase genes, including blaNDM+blaKPC, blaNDM+blaVIM, and blaNDM+blaOXA‐48. Phenotypic testing identified carbapenemase activity in 14 isolates, whereas six additional gene‐positive isolates were missed by mCIM. Phylogenetic analysis demonstrated close relatedness of detected carbapenemase genes to globally circulating variants.

**Conclusions:**

Carbapenem resistance in *P. aeruginosa* is substantial in Bangladesh and is driven predominantly by *blaNDM*. The detection of isolates carrying multiple carbapenemase genes highlights the growing complexity of antimicrobial resistance. Combining molecular and phenotypic approaches can improve surveillance, infection control, and antimicrobial stewardship in resource‐limited settings.

## 1. Introduction

*Pseudomonas aeruginosa* is a leading cause of nosocomial infections, particularly among patients with severe underlying illness or immunocompromise. The increasing prevalence of multidrug‐resistant *P. aeruginosa* poses a major clinical challenge, as these infections are associated with prolonged hospitalization, limited treatment options, and high mortality [1]. The organism′s intrinsic resistance to multiple antimicrobial classes, combined with its capacity to rapidly acquire additional resistance determinants, severely restricts effective therapy [2]. Consequently, carbapenems are frequently used as last‐resort agents for the treatment of resistant *P. aeruginosa* infections.

The widespread and often empiric use of these last‐line agents has driven the emergence of resistance, particularly in hospital settings. Carbapenem‐resistant *P. aeruginosa* (CRPA) is classified by the World Health Organization as a priority pathogen of critical concern, reflecting its substantial clinical and public health impact [3]. Despite reliance on polymyxins for the treatment of carbapenem‐resistant infections, clinical outcomes remain poor, and resistance to colistin is increasingly reported [4, 5].

Carbapenem resistance in *P. aeruginosa* is most commonly mediated by carbapenem‐hydrolyzing enzymes, including metallo‐*β*‐lactamases and oxacillinases.

The five major carbapenemase genes, NDM, IMP, VIM, KPC, and OXA‐48, encode *β*‐lactamase enzymes that can hydrolyze carbapenems and other *β*‐lactam antibiotics. These enzymes contribute to multidrug resistance among gram‐negative bacteria and reduce available treatment options. The genes represent the major Ambler carbapenemase classes. KPC belongs to Class A serine carbapenemases, NDM, VIM, and IMP belong to Class B metallo‐*β*‐lactamases, and OXA‐48‐like enzymes belong to Class D oxacillinases [6, 7].

These genes are often carried on plasmids and integrons, which facilitate horizontal transfer between bacterial species. They are widely distributed among Enterobacterales and nonfermenting gram‐negative bacteria, including *P. aeruginosa* and *Acinetobacter* spp., contributing to their global spread [6–11]. The presence of more than one carbapenemase gene in the same isolate has been increasingly reported and is associated with broader antimicrobial resistance and poorer clinical outcomes [10, 11]. Molecular methods such as multiplex PCR, real‐time PCR, lateral flow assays, and CARBA‐5 are commonly used for the detection of these genes. These methods support infection prevention, infection control, and antimicrobial stewardship programs [6, 8, 12, 13].

Although phenotypic resistance to carbapenems and colistin is increasingly documented, data linking resistance phenotypes to specific genetic determinants in *P. aeruginosa* remain limited in many low‐ and middle‐income countries (LMICs), including Bangladesh. In particular, information on the coexistence of carbapenemase genes within the same clinical isolates is scarce. Molecular epidemiological data from Bangladesh are especially limited. Therefore, this study is aimed at determining the prevalence of carbapenem resistance and coexistence of carbapenemase genes among clinical *P. aeruginosa* isolates from a tertiary‐care hospital in Chattogram, Bangladesh, and characterizing the underlying genetic basis of resistance using targeted PCR and Sanger sequencing. By integrating phenotypic susceptibility testing with focused molecular analysis, this study provides new insights into the molecular landscape of last‐line antimicrobial resistance in *P. aeruginosa* in a resource‐limited setting.

## 2. Materials and Methods

### 2.1. Study Design

This is a cross‐sectional observational study.

### 2.2. Study Period and Population

The study was done from January 2023 to December 2023 and the samples were collected from admitted patients in the Departments of Medicine, Surgery, Gynaecology and Obstetrics, Orthopedics, Urology, and Pediatrics of Chittagong Medical College Hospital.

### 2.3. Sample Size

The minimum sample size was calculated using the formula *n* = *Z* ^2^ *p* *q*/*d* ^2^, assuming a 95% confidence interval (*Z* = 1.96), a 5% margin of error (*d* = 0.05), and an expected prevalence (*p*) of carbapenem resistant *Pseudomonas* spp. is 26.8% [14] from a previous study conducted in Bangladesh that included urine, sputum, endotracheal aspirate (ETA), wound swab, and blood specimens. The calculated sample size was 303. Accounting for a 10% potential nonresponse or incomplete data rate, the target sample was adjusted to 333. A total of 335 specimens were included in this study, exceeding the minimum requirement. Written informed consent was obtained from all participants. The sampling technique was nonprobability purposive sampling. The sample size was calculated according to our previously described [15] formula.

### 2.4. Eligibility Criteria

#### 2.4.1. Inclusion Criteria

*Pseudomonas* spp. isolated from patients with suspected urinary tract infection, wound infection, respiratory tract infection, or febrile illness, as well as patients in mechanical ventilation in intensive care unit (ICU) were included, irrespective of age, gender, or antibiotic intake.

#### 2.4.2. Exclusion Criteria

Isolates other than *Pseudomonas* spp. were excluded.

### 2.5. Microbiological Methods

Microbiological methods were followed standard protocols and culture procedures, and PCR methods followed our previous [15] work.

#### 2.5.1. Sputum Culture

Early morning sputum specimens were collected before food or fluid intake. Patients were instructed to rinse their mouths with water and expectorate deep sputum into sterile, tightly capped containers [16]. Gram‐stained smears were examined for specimen quality. Samples containing more than 25 neutrophils per low‐power field (LPF) and fewer than 10 squamous epithelial cells per LPF were considered acceptable for culture. Specimens with 10 or more squamous epithelial cells per LPF were rejected [16]. Semiquantitative culture was performed using a calibrated 10 *μ*L loop. Colony counts were categorized as mild (≤ 10^3^ CFU/mL), moderate (10^4^ CFU/mL), or heavy (≥ 10^5^ CFU/mL). Growth extending to the second or subsequent quadrants of the culture plate was considered significant.

#### 2.5.2. Urine Culture

Midstream clean‐catch urine specimens were collected in sterile containers after appropriate cleansing procedures for male, female, and catheterized patients. Direct microscopic examination was performed using uncentrifuged urine samples. Urine culture was carried out with a calibrated 1 *μ*L loop, and bacterial growth of ≥ 10^5^ CFU/mL was considered significant [16].

#### 2.5.3. ETA Culture

ETA specimens were collected aseptically from mechanically ventilated ICU patients and transported immediately to the microbiology laboratory. Gram‐stained smears were examined for specimen quality, and samples containing > 10 squamous epithelial cells per LPF were rejected [16]. Semiquantitative culture was performed using a calibrated 10 *μ*L loop. Growth in the second or subsequent quadrants (≥ 10^4^ CFU/mL) was considered significant [16].

#### 2.5.4. Wound Swab Culture

Wound swab specimens were collected aseptically from the deeper areas of cleaned wounds using sterile cotton‐tipped swabs and transported promptly to the laboratory. Gram staining was performed, and specimens were cultured by standard inoculation and streaking techniques on appropriate culture media [16].

#### 2.5.5. Blood Culture

Blood specimens were collected under aseptic conditions following skin disinfection with 70% alcohol. Ten milliliters of blood from adults, or age‐appropriate volumes from children, were inoculated into BD BACTEC Plus Aerobic/F culture bottles. The bottles were incubated according to standard procedures, and after positive signal, subcultures were performed daily for up to 5 days. Subcultures were inoculated onto blood agar and MacConkey agar plates [16].

#### 2.5.6. Identification of Organisms

Bacterial isolates were identified based on colony morphology, pigmentation, hemolytic characteristics, Gram staining results, and standard biochemical tests, including catalase, oxidase, triple sugar iron (TSI), citrate utilization, motility, indole, and urease tests [16].

#### 2.5.7. Antimicrobial Susceptibility Testing

Antimicrobial susceptibility testing was performed using the modified Kirby–Bauer disc diffusion method on Mueller–Hinton agar. Antibiotic discs (Oxoid, United Kingdom) were stored at −20°C, and quality control was performed using *Escherichia coli* ATCC 25922 [16]. Colistin susceptibility was determined using the colistin agar test (CAT) according to established procedures [17].

#### 2.5.8. Phenotypic Detection of Carbapenemase Production

Carbapenemase production was screened using the modified carbapenem inactivation method (mCIM) following CLSI M100 (2023) guidelines [17]. A 10 *μ*L loopful of the test organism was suspended in 2 mL of tryptic soy broth (TSB) and mixed thoroughly by vortexing for 15 s. A 10 *μ*g meropenem disc was placed in the suspension and incubated at 35°C for 4 h. Simultaneously, a 0.5 McFarland suspension of *Escherichia coli* ATCC 25922 was prepared and inoculated onto Mueller–Hinton agar to create a lawn culture. After incubation, the meropenem disc was removed from the test suspension and placed onto the inoculated agar surface. The plate was then incubated overnight at 37°C [17].

Interpretation of results was performed according to CLSI recommendations [17]. An inhibition zone of 6 to 15 mm, or the presence of colonies within a zone measuring 16 to 18 mm, was considered positive for carbapenemase production. A zone diameter of 19 mm or greater was considered negative. A zone diameter of 16 to 18 mm without colonies was interpreted as indeterminate. *Klebsiella pneumoniae* ATCC BAA‐1705 was used as the positive control strain.

#### 2.5.9. Molecular Methods

##### 2.5.9.1. DNA Extraction

Bacterial isolates were subcultured on MacConkey agar and subsequently inoculated into TSB. Following incubation, the cultures were centrifuged and genomic DNA was extracted using the Monarch Genomic DNA Purification Kit according to the manufacturer′s instructions. The extracted DNA was stored at −20°C until further analysis.

##### 2.5.9.2. Multiplex PCR Detection of Resistance Genes

Multiplex PCR was used to detect carbapenemase genes (*blaNDM*, *blaVIM*, *blaKPC*, *blaOXA-48*, and *blaIMP*). Reaction: 25 *μ*L (12.5 *μ*L master mix, 1 *μ*L forward primer, 1 *μ*L reverse primer, 2 *μ*L DNA template and the remaining volume was adjusted with nuclease‐free water). Negative control (nuclease‐free water) was used in each PCR run to exclude contamination, but no positive control was used because of resource constraints. Primer sequences were verified using Primer‐BLAST by the National Center for Biotechnology Information (NCBI), and primers were synthesized commercially by Macrogen Inc., Seoul, South Korea. PCR products were visualized on 2% agarose gels. Primer sequences and amplicon sizes are listed in Table 1.

**Table 1:** Primers used for detection of *P. aeruginosa*, carbapenemase genes.

Target	Sequences (5 ^′^‐3 ^′^)	Product length (bp)
16S rDNA of *P.aeruginosa*	F‐GACGGGTGAGTAATGCCTA	617
	R‐CACTGGTGTTCCTTCCTATA	
*blaIMP*	F‐GGAATAGAGTGGTGCTTAAYTCTC	232
	R‐GGTTTAAYAAAACAACCACC	
*blaVIM*	F‐GATGGTGTTTGGTCGCATA	390
	R‐CGAATGCGCAGCACCAG	
*blaNDM*	F‐GGTTTGGCGATCTGGTTTTC	621
	R‐CGGAATGGCTCATCACGATC	
*blaKPC*	F‐CGTCTAGTTCTCTGCTGTCTTG	798
	R‐CTTGTCATCCTTGTTAGGCG	
*blaOXA-48*	F‐GCGTGGTTAAGGATGAACAC	440
	R‐CATCAAGTTCAACCCAACCG	

The PCR conditions were as follows:

• For 16S rDNA, blaVIM, blaKPC, and blaOXA‐48: initial denaturation at 95°C for 3 min; 40 cycles of denaturation at 95°C for 1 min, annealing at 56°C for 40 s, and extension at 72°C for 1 min; final extension at 72°C for 5 min.
• For blaNDM: initial denaturation at 95°C for 5 min; 36 cycles of denaturation at 95°C for 1 min, annealing at 57°C for 40 s, and extension at 72°C for 1 min; final extension at 72°C for 10 min.
• For blaIMP: initial denaturation at 95°C for 5 min; 36 cycles of denaturation at 95°C for 1 min, annealing at 54°C for 40 s, and extension at 72°C for 1 min; final extension at 72°C for 5 min.

##### 2.5.9.3. Automated Sanger Sequencing of Cocarriage Carbapenemase Genes

To further characterize the cocarriage of carbapenemase determinants, Sanger sequencing was selectively performed on PCR amplicons representing the carbapenemase genes detected in the three isolates exhibiting cocarriage (isolates 17, 160, and 209). Thus, sequencing was intended as a targeted confirmation and phylogenetic characterization of cocarriage profiles rather than comprehensive sequencing of all PCR‐positive isolates. Amplicons were purified and sequenced (Macrogen, Korea) using the same forward primers as in PCR. Sequence data were analyzed and trimmed with BioEdit v7.1.3. Phylogenetic trees were constructed using MEGA XII software with the neighbor‐joining method, 1000 bootstrap replicates, and the Kimura 2‐parameter model; evolutionary distances were calculated using the maximum composite likelihood method. Sequence homology was confirmed through NCBI BLAST analysis.

#### 2.5.10. Statistical Analysis

Data were managed in Microsoft Excel and analyzed using IBM SPSS v25. Associations between categorical variables were assessed with Fisher′s exact test. Associations between categorical variables were not assessed, but descriptive observation was stated, because of the small sample size. Given the limited number of meropenem‐resistant isolates, multivariable logistic regression was not performed to avoid model overfitting. Two‐sided *p* values < 0.05 were considered statistically significant. Missing data were handled by casewise deletion, and Bonferroni correction was applied for multiple comparisons. Graphs were generated using Excel and SPSS.

## 3. Result

### 3.1. Identification of *P. aeruginosa*

Of the 335 clinical specimens processed, 52 *Pseudomonas* spp. isolates were recovered, of which 48 (92.3%) were confirmed as *P. aeruginosa* by 16S rDNA PCR and included in the final analysis.

### 3.2. Antimicrobial Susceptibility

Among the 48 PCR‐confirmed *P. aeruginosa* isolates, 13 (27.1%) were resistant to meropenem and 10 (20.8%) to imipenem. Aminoglycoside resistance was detected in 16 (33.3%) isolates for amikacin and 18 (37.5%) for gentamicin, whereas levofloxacin resistance was found in 18 (37.5%). Among *β*‐lactams, resistance rates were 41.7% (20) for cefepime, 47.9% (23) for ceftazidime, 22.9% (11) for aztreonam, and 20.8% (10) for piperacillin‐tazobactam (Figure 1). CAT revealed seven isolates (14.6%) were classified as resistant (MIC ≥ 4 *μ*g/mL), whereas the remainder were intermediate.

**Figure 1:**
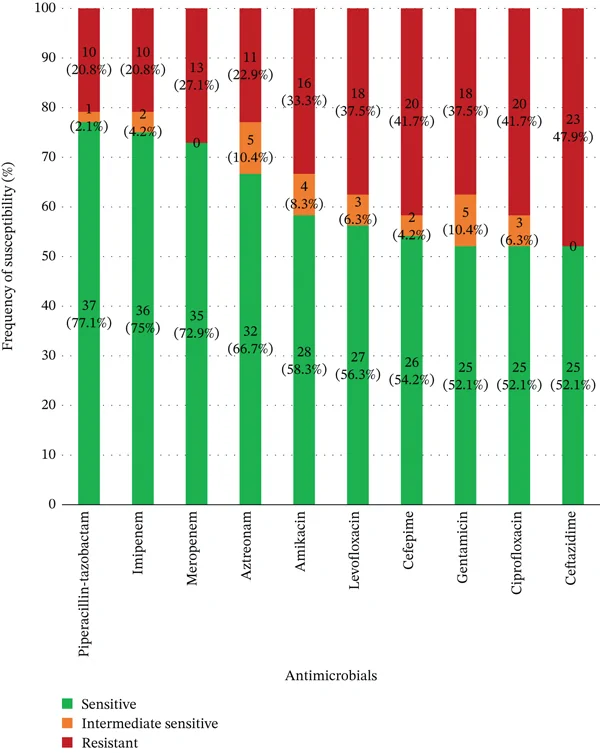
Antimicrobial resistance pattern of *P. aeruginosa* isolates (*n* = 48).

### 3.3. Carbapenem Resistance Genes

PCR detected carbapenemase genes in 20 isolates (41.7%). Single carbapenemases included *blaNDM* (11, 22.9%), *blaVIM* (4, 8.3%), *blaKPC* (1, 2.1%), and *blaOXA-48* (1, 2.1%). Co‐occurring carbapenemases were observed in three isolates: *blaNDM*+*blaOXA-48*, *blaNDM*+*blaKPC*, and *blaNDM*+*blaVIM*.

### 3.4. Phenotypic–Genotypic Correlation

Phenotypic carbapenemase activity (mCIM) was positive in 14 isolates (29.2%). All mCIM‐positive isolates carried at least one carbapenemase gene, whereas six mCIM‐negative isolates (12.5%) were genotypically positive, indicating silent carriers (Table 2).

**Table 2:** Comparison of mCIM and multiplex PCR for detection of carbapenemase genes among *P. aeruginosa* isolates (*n* = 48).

mCIM	PCR positive	PCR negative	Total	McNemar′s exact *p* = 0.031
Positive	14 (29.2%)	0 (0.0%)	14 (29.2%)	Sensitivity = 70.0*%*
				Specificity = 100*%*
Negative	6 (12.5%)	28 (58.3%)	34 (70.8%)	PPV = 100*%*
				NPV = 82.4*%*
Total	20 (41.7%)	28 (58.3%)	48 (100%)	Overall agreement = 87.5*%*

### 3.5. Multiple Antibiotics Resistance

MAR indices for carbapenemase‐positive isolates ranged from 0.14 to 1.00, reflecting high multidrug resistance. Detailed resistance phenotypes and carbapenemase profiles are shown in Table 3.

**Table 3:** Multiple antibiotics resistant index (MAR) of carbapenemase gene identified *P. aeruginosa* (*n* = 20).

ID	Number of resistant drug category (a)	Number of tested drug category (b)	MAR index (a/b)	Carbapenemase gene detected	Carbapenem susceptibility	
					Meropenem	Imipenem
7	5	7	0.71	VIM	R	R
15	3	7	0.43	NDM	R	R
17	6	7	0.86	NDM+KPC	R	R
51	3	7	0.43	NDM	S	S
54	5	7	0.71	OXA‐48	R	S
60	3	7	0.43	NDM	S	S
111	4	7	0.57	VIM	R	R
112	3	7	0.43	NDM	R	IS
115	6	7	0.86	NDM	S	S
151	2	7	0.29	NDM	R	IS
155	1	7	0.14	NDM	S	S
160	7	7	1	NDM+VIM	R	R
170	1	7	0.14	NDM	R	S
209	4	7	0.57	NDM+OXA‐48	R	R
215	2	7	0.29	VIM	R	R
227	3	7	0.43	NDM	S	S
245	4	7	0.57	KPC	R	R
250	4	7	0.57	VIM	R	R
275	4	7	0.57	NDM	S	R
294	4	7	0.57	NDM	S	S

*Note:* a is the fraction of the quantity of antimicrobials which an isolate is resistant to; b is the sum quantity of antimicrobials to which the isolates were used against.

Abbreviations: IS, intermediate sensitive; R, resistant; S, susceptible.

### 3.6. Factors Associated With Carbapenem Resistance

In our study, meropenem resistance appeared more frequent among isolates from ICU patients, patients with diabetes, and ETA specimens. However, because only 13 isolates were meropenem‐resistant, multivariable modeling was not considered sufficiently reliable and these associations should be interpreted cautiously.

### 3.7. Sanger Sequencing and Phylogenetic Analysis

Partial sequencing was performed for carbapenemase genes from isolates with coexisting resistance determinants (three *blaNDM* and one each of *blaKPC*, *blaVIM*, and *blaOXA-48*; isolate IDs 17, 160, and 209). Sequences were submitted to NCBI GenBank (accession numbers: *blaNDM* PX172128.1, PX349204.1, PX378096.1; *blaVIM* PX378094.1; *blaOXA-48* PX378095.1; *blaKPC* PX404855.1).

Phylogenetic analysis using neighbor‐joining trees demonstrated clustering of study isolates with globally circulating carbapenemase variants (Figure 2).

**Figure 2:**
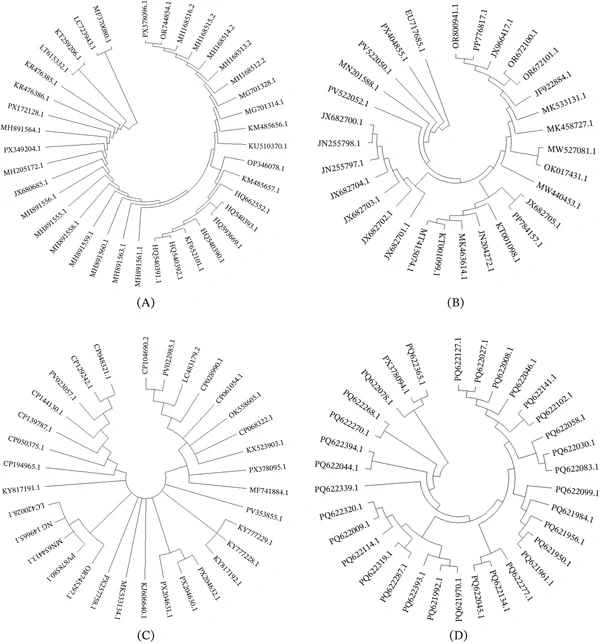
Phylogenetic tree of (A) NDM, (B) KPC, (C) OXA‐48, and (D) VIM by N‐J method.

## 4. Discussion

This study demonstrates high concordance between phenotypic identification and PCR confirmation of *P. aeruginosa*, with 92.3% of presumptive isolates molecularly verified, consistent with reports from Iran, Brazil, and Egypt [18–20]. Variability across studies reflects differences in specimen selection, laboratory capacity, and diagnostic algorithms, challenges amplified in LMICs [21–23].

Antimicrobial susceptibility testing revealed moderate resistance to carbapenems [21–28]), aminoglycosides, fluoroquinolones, and cephalosporins, comparable with reports from the Arabian Gulf and Saudi Arabia [24–28]. Colistin resistance was detected in 14.6% of isolates, higher than global averages but similar to other Asian regions [29]. Heteroresistance among carbapenem‐resistant isolates highlights the limitations of routine diagnostics and potential for treatment failure.

Molecular analysis identified carbapenemase genes in 41.7% of isolates, predominantly *blaNDM*, with some isolates coharboring multiple carbapenemases, reflecting global dissemination and increased therapeutic complexity [28, 30–36].

An important finding was the detection of carbapenemase genes in isolates that remained phenotypically susceptible to carbapenems. Several blaNDM‐positive isolates, including isolates 51, 60, 115, 227, and 294, were susceptible to both meropenem and imipenem. Such genotype–phenotype discordance has been described and may reflect differences in gene expression, promoter activity, gene copy number, genetic context, or other bacterial resistance mechanisms that influence the phenotypic expression of carbapenem resistance. However, these mechanisms were not investigated in the present study, and therefore, no specific explanation can be assigned to the individual isolates. The finding nevertheless highlights that carriage of a carbapenemase gene does not invariably translate into phenotypic carbapenem resistance and supports the complementary use of molecular and phenotypic methods. Phenotypic carbapenemase detection using the modified mCIM showed high specificity but missed several gene‐positive isolates, highlighting the need for combined phenotypic and molecular diagnostics in LMIC settings [37, 38].

In present study, carbapenem resistance appeared more frequent among isolates obtained from ICU patients, patients with diabetes, and ETA specimens. However, the small number of resistant isolates precluded reliable multivariable modeling, and these observations should therefore be interpreted as exploratory rather than independent predictors. ICU stay and invasive respiratory procedures are well‐recognized risk factors for CRPA because of prolonged hospitalization, device use, and broad‐spectrum antibiotic exposure [39–42]. Diabetes may contribute to acquisition or selection of resistant strains, reflecting comorbidity burden [41, 42]. Respiratory isolates, particularly from ETA, represented a key reservoir for CRPA, consistent with regional and global data [31, 40].

In conclusion, carbapenem resistance in *P. aeruginosa* in Bangladesh is substantial and driven predominantly by *blaNDM*. Integration of molecular diagnostics with phenotypic testing enhances surveillance accuracy and informs empiric therapy and stewardship in resource‐limited settings. These findings contribute critical LMIC data to the WHO Global Antimicrobial Resistance Surveillance System and underscore the need for coordinated One Health strategies to preserve the efficacy of last‐line antimicrobials.

## 5. Strength and Limitations

• This study combined phenotypic testing, multiplex PCR, Sanger sequencing, and phylogenetic analysis to characterize carbapenem resistance in clinical *P. aeruginosa* isolates.
• The study provides novel data on the cocarriage of major carbapenemase genes among *P. aeruginosa* isolates in Bangladesh.
• Clinical and microbiological data were integrated to identify independent predictors of carbapenem resistance.
• The study was conducted in a single tertiary‐care hospital with a relatively small sample size, which may limit generalizability.
• Sanger sequencing was limited to selected amplicons from the three isolates exhibiting carbapenemase gene cocarriage. Therefore, the sequence‐based findings should not be interpreted as representative of all 20 carbapenemase gene‐positive isolates.
• Only selected carbapenemase genes were investigated, and whole‐genome sequencing was not performed, potentially missing other resistance mechanisms and transmission pathways.

## Author Contributions

P.D. conceptualized the study. P.D. and S.K.C. designed the methodology and formal analysis. All clinical and laboratory procedures were conducted by P.D. and M.F.U. and technical staff. S.B., P.M., and A.A.K. were responsible for data curation. Data validation was led by S.K.C., who contextualized the findings based on local antimicrobial prescribing practices, infection control challenges, and regional resistance patterns. P.D. and S.N. wrote the original draft, which is reviewed and edited by S.K.C. and A.A.K.

## Funding

No funding was received for this manuscript.

## Ethics Statement

Ethical approval was granted by the Ethical Review Committee of Chittagong Medical College (PG.009.2022/905).

## Conflicts of Interest

The authors declare no conflicts of interest.

## Supporting information


**Supporting Information** Additional supporting information can be found online in the Supporting Information section. File S1: STROBE statement‐checklist of items that should be included in reports of cross‐sectional studies.

## Data Availability

Raw data are available from the corresponding author upon reasonable request. Sequence data for study isolates have been deposited in the NCBI GenBank database under the accession numbers provided in the manuscript.
