# Supplementary material for: Molecular Characterization of Cocarriage Carbapenemase Genes in Clinical Pseudomonas aeruginosa
Source: Int J Microbiol. 2026 Oct 2;2026:5679086. doi: 10.1155/ijm/5679086 (PMC13633459; doi:10.1155/ijm/5679086)
Supplement: Supplementary file 1 [file IJM-2026-5679086-s001.doc]

STROBE Statement-Checklist of items that should be included in reports of ***cross-sectional studies***

|  | Recommendation |
| --- | --- |
| **Title and abstract** | (*a*) Indicating the study’s design with a commonly used term in the title or the abstract |
|  | (*b*) Providing in the abstract an informative and balanced summary of what was done and what was found |
| Background/rationale | Explaining the scientific background and rationale for the investigation being reported |
| Objectives | Stated the specific objectives |
| Study design | Presenting the key elements of study design early in the paper |
| Setting | Describing the setting, locations, and relevant dates, including periods of recruitment, exposure and data collection |
| Participants | (*a*) Giving the eligibility criteria, and the sources and methods of selection of participants |
| Variables | Clearly defining the all outcomes, exposures, predictors, potential confounders, and effect modifiers. |
| Bias | Describing any efforts to address potential sources of bias |
| Study size | Explaining how the study size was arrived |
| Quantitative variables | Explaining how quantitative variables were handled in the analyses. |
| Statistical methods | (*a*) Describing all statistical methods, including those used to control for confounding |
|  | (*b*) Describing the methods used to examine subgroups and interactions |
|  | (*c*) Explaining how missing data were addressed |
|  | (*d*) describing the analytical methods taking account of sampling strategy |
|  | (*e*) Describing any sensitivity analyses |
| Participants | (a) Reporting the numbers of individuals at each stage of study—eg numbers potentially eligible, examined for eligibility, confirmed eligible, included in the study, completing follow-up, and analysed |
|  | (b) Giving the reasons for non-participation at each stage |
| Descriptive data | (a) Giving the characteristics of study participants (eg demographic, clinical, social) and information on exposures and potential confounders |
|  | (b) Indicating the number of participants with missing data for each variable of interest |
| Outcome data | Reporting the numbers of outcome events were done |
| Main results | (*a*) Giving the adjusted estimates and their precision (eg, 95% confidence interval). |
|  | (*b*) Reporting of category boundaries when continuous variables were categorized |
| Other analyses | Reporting of other analyses was done |
| Key results | Summarising the key results with reference to study objectives |
| Limitations | Discussing the limitations of the study, taking into account sources of potential bias or imprecision. Discuss both direction and magnitude of any potential bias |
| Interpretation | Giving the interpretation of results considering objectives, limitations, multiplicity of analyses, results from similar studies, and other relevant evidence |
| Generalisability | Discussing the generalisability of the study results in manuscript |
| Funding | No external funding received |
